# Receptor-Type Protein-Tyrosine Phosphatase ζ and Colony Stimulating Factor-1 Receptor in the Intestine: Cellular Expression and Cytokine- and Chemokine Responses by Interleukin-34 and Colony Stimulating Factor-1

**DOI:** 10.1371/journal.pone.0167324

**Published:** 2016-11-29

**Authors:** Stephanie Zwicker, Daniela Bureik, Madeleen Bosma, Gisele Lago Martinez, Sven Almer, Elisabeth A. Boström

**Affiliations:** 1 Department of Dental Medicine, Division of Periodontology, Karolinska Institutet, Huddinge, Sweden; 2 Department of Cell and Molecular Biology, Karolinska Institutet, Stockholm, Sweden; 3 Department of Medicine, Solna, Karolinska Institutet, Stockholm, Sweden; 4 GastroCentrum, Karolinska University Hospital, Solna, Stockholm, Sweden; Midwestern University, UNITED STATES

## Abstract

Differential intestinal expression of the macrophage growth factors colony stimulating factor-1 (CSF-1), interleukin (IL)-34, and their shared CSF-1 receptor (CSF-1R) in inflammatory bowel disease (IBD) has been shown. Diverse expression between CSF-1 and IL-34, suggest that IL-34 may signal via an alternate receptor. Receptor-type protein-tyrosine phosphatase ζ (PTPRZ1, RPTP-ζ), an additional IL-34 receptor, was recently identified. Here, we aimed to assess *PTPRZ1* expression in IBD and non-IBD intestinal biopsies. Further, we aimed to investigate cellular PTPRZ1 and CSF-1R expression, and cytokine- and chemokine responses by IL-34 and CSF-1. The expression of *PTPRZ1* was higher in non-IBD colon compared to ileum. *PTPRZ1* expression was not altered with inflammation in IBD, however, correlated to *IL34*, *CSF1*, and *CSF1R*. The expression patterns of PTPRZ1 and CSF-1R differed in peripheral blood mononuclear cells (PBMCs), monocytes, macrophages, and intestinal epithelial cell line. PBMCs and monocytes of the same donors responded differently to IL-34 and CSF-1 with altered expression of tumor-necrosis factor α (TNF-α), IL-1β, interferon γ (IFN-γ), IL-13, IL-8, and monocyte chemotactic protein-1 (MCP-1) levels. This study shows that *PTPRZ1* was expressed in bowel tissue. Furthermore, CSF-1R protein was detected in an intestinal epithelial cell line and donor dependently in primary PBMCs, monocytes, and macrophages, and first hints also suggest an expression in these cells for PTPRZ1, which may mediate IL-34 and CSF-1 actions.

## Introduction

Crohn’s disease (CD) and ulcerative colitis (UC) are chronic inflammatory conditions of the gastrointestinal tract that are collectively referred to as inflammatory bowel disease (IBD). Although the pathogenesis of IBD remains incompletely understood, it involves a combination of genetic disposition, environmental factors, and dysregulation of the immune system [1–3].

Cells of the mononuclear lineage including monocytes and monocyte-derived macrophages increase in numbers with inflammation in IBD, where resident tissue macrophages play a central role in intestinal homeostasis as they phagocytose invading pathogens which penetrate the epithelial barrier [4]. Binding of colony-stimulating factor 1 (CSF-1, M-CSF), to its CSF-1 receptor (CSF-1R) promotes macrophage differentiation and viability, via the activation of the transcription factor PU.1 [5]. In 2008, another functionally overlapping ligand of CSF-1R was identified: interleukin-34 (IL-34) [6]. Because of high structural similarities, IL-34 and CSF-1 compete for binding to their shared receptor CSF-1R, where IL-34 binds more tightly compared to CSF-1, while both induce proliferation and cell survival in monocytes (reviewed in [7]). Recently, our group and Franzé et al., reported regulation of IL-34 and CSF-1 with inflammation in IBD [8, 9]. We also reported different expression patterns of IL-34 and CSF-1 in ileum and colon of non-IBD subjects, higher CSF-1R expression in sigmoid compared to transverse colon, and up-regulated CSF-1R expression with inflammation in IBD patients [8]. In addition to the gut, CSF-1R is detected in other tissues including the brain, liver and heart [10]. An additional receptor of IL-34, receptor-type protein-tyrosine phosphatase ζ (PTPRZ1, RPTP-ζ), was recently identified by Nandi and colleagues who showed signalling through PTPRZ1 by IL-34, but not CSF-1, in mouse brain and in the human glioblastoma cell line U251 [11]. PTPRZ1 is a constitutively activated phosphatase, where binding of IL-34 inhibits the phosphatase activation, followed by a downstream tyrosine phosphorylation. PTPRZ1 is expressed in different tissues including the brain, stomach, and small intestine, according to the human protein atlas database [10]. Interestingly, inhibition of PTPRZ1 was suggested as a treatment for Parkinson’s disease based on delayed recovery from demyelinating lesions in a mouse model lacking PTPRZ1 [12, 13]. The PTPRZ1 knockout mice have deficiencies in learning and memory, but no other abnormalities are described [14]. The expression and regulation of PTPRZ1 in the gut has not been characterized in detail to date.

Macrophage growth factors are implicated in several inflammatory conditions including Sjögren’s syndrome where IL-34 expression is increased in the salivary glands, and in rheumatoid arthritis (RA) where IL-34 is increased in serum and synovial fluid [15, 16]. IL-34 has been suggested as a potential local therapeutic target, since tumor-necrosis factor α (TNF-α) blockade by Infliximab in RA patients resulted in decreased IL-34 expression [17]. Furthermore, blocking IL-34 in colon explants from IBD patients was shown to decrease TNF-α and IL-6 expression [9]. On cellular level, IL-34 is induced by TNF-α in fibroblasts [16, 18] and in gut epithelial cells through activation of the nuclear factor kappa B (NF-κB) [8]. Eda and colleagues reported that IL-34 and CSF-1 induce cytokines and chemokines in human whole blood, and induction of the cc-chemokine ligand 20 (CCL20) was detected after IL-34 stimulation in colon epithelial cells through phosphorylation of ERK1/2 and JNK [19, 20]. Little is known, however, about the capacity of IL-34 and CSF-1 to regulate cytokines and chemokines in specific immune cells such as PBMCs, monocytes and macrophages, and in non-immune cells.

The aims of the present study were, (i) to assess the intestinal expression of PTPRZ1 in non-IBD subjects and in patients with IBD, (ii) to assess the cellular CSF-1R and PTPRZ1 expression, and, (iii) to evaluate the cytokine- and chemokine responses following IL-34 and CSF-1 stimulation of intestinal epithelial cells, PBMCs, monocytes, and macrophages. Together, our findings indicate diverse functions of macrophage growth factors depending on the cell type, which in turn point to possibilities to investigate IL-34 and CSF-1 as specific cell type-dependent targets in IBD.

## Materials and Methods

### Human inflammatory bowel disease cohort

45 adult patients under investigation for suspected bowel diseases or known IBD and 27 patients without IBD and without intestinal inflammation or any pathological findings were subjected to colorectal and ileal mucosal biopsies during routine endoscopy (S1 Table)[8]. Each biopsy was classified by one experienced endoscopist (S.A.) as ‘inflamed’ or ‘non-inflamed’ based on evaluations of the macroscopic findings and histopathologic assessment from biopsies collected in parallel to, and from the same locations. Biopsies collected for RNA purification were immersed in RNA *later* RNA stabilization reagent (Qiagen, Hilden, Germany) and stored at 4°C overnight and thereafter at -20°C until RNA purification. cDNA from 24 non-IBD and 21 IBD patients was available for this study. The study was carried out in accordance with the Declaration of Helsinki (2008) of the World Medical Association and approved by the Regional Ethical Review Board in Linköping, Sweden (Dnr 2011/201-31). All participants gave their written informed consent.

### PBMC, monocyte and macrophage cultures

Peripheral blood mononuclear cells (PBMCs) were isolated from buffy-coated blood of healthy blood donors using Ficoll-Hypaque gradient centrifugation (BD Diagnostics, Franklin Lakes, NJ, USA). To isolate monocytes the EasySep Human monocyte enrichment kit without CD16 depletion was used according to manufacturer’s protocols (StemCell Technologies, Vancouver, Canada). PBMCs and monocytes were seeded into 6-well plates and incubated in absence (control) used as controls, or presence of IL-34 or CSF-1 (10 and 50 ng/ml, BioLegend, San Diego, CA, USA) for 1, 6 and 24 h. CSF-1R blocking antibody or IgG1 control (50 ng/ml, R&D Systems, Minneapolis, MN, USA) were added together with IL-34 or CSF-1 and incubated for 6 and 24 h.

Macrophages were generated at day 8 from 5x10^5^ monocytes plated per well in 6 well plates with complete RPMI media supplemented with 50 ng/ml CSF-1 or IL-34 (Biolegend, San Diego, CA, USA). The cells were polarized on day 8 with LPS/IFN-γ (50 ng/ml, BioLegend, San Diego, CA, USA) or IL-4/IL-13 (50 ng/ml, BioLegend, San Diego, CA, USA) into M1 or M2-like monocyte derived macrophages, respectively, for 24 h, and non-polarized monocyte derived macrophages were used as controls.

### Colon epithelial cells

Caco-2 cells (American Type Culture Collection, Rockville, MD, USA) were cultured in DMEM supplemented with 10% FBS (Gibco-Brl/Life Technologies, Paisley, UK), 1% NEEA (Gibco-Brl/Life Technologies, Paisley, UK), and 1% Glutamax (Invitrogen, Carlsbad, CA, USA) at 37°C and 5% CO_2_. Cells were seeded in 6- or 24-well plates and after attachment for 48 hours the media was changed and the cells were incubated in the absence (controls) or presence of IL-34 and/or CSF-1 (10 ng/ml, BioLegend, San Diego, CA, USA) for 6 hours. Cell lysates from 24-well plate were subjected to RNA-isolation and cell lysates and supernatants from 6 well plates were subjected to protein analysis.

### RNA-isolation, cDNA synthesis, and quantitative real-time polymerase chain reaction (q-PCR)

Biopsy specimens for RNA were homogenized using a TissueRuptor and disposable probes. RNA was purified using the AllPrep DNA/RNA mini kit (Qiagen, Hilden, Germany) according to the manufacturer’s instructions. RNasin Plus RNase inhibitor (Promega Corporation, Madison, WI, USA) was added to the RNA. Two preparations of 2 μg RNA from each biopsy were reverse transcribed in a volume of 20 μl each using the High Capacity cDNA Reverse Transcription Kit with RNase inhibitor (Applied Biosystems, Foster City, CA, USA), according to the manufacturer’s instructions. For each biopsy, the resulting cDNA libraries were pooled and stored at -80°C. q-PCRs on the cDNA from the biopsies was performed using iTaq Universal SYBR green supermix (Life Technologies, Stockholm, Sweden) on a ViiA7 Real-Time PCR System (Life Technologies, Stockholm, Sweden). From Caco-2 cells, PBMCs, monocytes and macrophages total RNA was isolated using the Quick-RNA MiniPrep kit (Zymo Research Corp, Irvine, CA, USA) and reversed transcribed by the High Capacity cDNA Reverse Transcription Kit (Applied Biosystems, Foster City, CA, USA) according to the manufacturer’s instructions. SYBR Green (Bio-Rad Laboratories, Hercules, CA, USA) in the 7500-fast-real-time detection system (Applied Biosystems, Foster City, CA, USA) was used to detect the mRNA levels of *IL1B*, *TNFA*, *IFNG*, *IL10*, *IL13*, *IL8*, *MCP1* (S2 Table) by specific primers (Eurofins, Ebersberg, Germany) related to the housekeeping gene *GAPDH* by the ΔΔCt method. To rule out the possibility of DNA contamination, samples in which the reverse transcription reaction had been omitted were also subjected to the PCR reaction, yielding no amplification. Primer sequences are shown in S2 Table.

### Immunoblot

Proteins were isolated from PBMCs, monocytes, macrophages and Caco-2 using T-PER Tissue Protein extraction lysis buffer (Thermo Fisher Scientific, Bonn, Germany) supplemented with cOmplete Protease Inhibitor Cocktail (Roche, Bromma, Sweden). 10 μg (PTPRZ1) and 15 μg (CSF-1R) total protein per well were separated using a 4–20% Mini-PROTEAN® TGX™ Precast Protein Gel (Bio-Rad Laboratories, Inc., Hercules, CA, USA) and transferred to nitrocellulose membranes (0.45 μM pore, GE Healthcare Life Science, Uppsala, Sweden). Membranes were blocked for 1 hour at room temperature on a shaker, in 3% ECL Prime blocking Reagent (GE Healthcare Life Science, Uppsala, Sweden) in TBS-T, or 5% milk in TBS-T for PTPRZ1 or CSF-1R, respectively. Subsequently membranes were incubated with anti-CSF-1R (Cell Signaling Technology, MA, USA) and anti-PTPRZ1 (Abcam, Cambridge, UK) in 3% ECL blocking reagent or 5% BSA/TBS-T at 4°C overnight.

Equal gel loading was confirmed by detection of the β-actin signal (Cell Signaling Technology, MA, USA). After washing steps with TBS-T, blots were incubated with horseradish-peroxidase-conjugated secondary antibodies (Cell Signaling Technology, MA, USA) for 1 h at room temperature and developed with Amersham ECL select Western Blotting Detection Reagent (GE Healthcare Life Science, Uppsala, Sweden) and visualized using ChemiDoc™ XRS (Bio-Rad Laboratories, Inc., Hercules, CA, USA).

### Enzyme-linked immunosorbent assay (ELISA)

Supernatants from PBMCs, monocytes, macrophages and Caco-2 cell stimulated with IL-34 and/or CSF-1 for 24 h were collected and IL-1β, IL-10 and MCP-1 were detected by ELISA according to the manufacturer’s protocol (Duo Set, R&D Systems, Minneapolis, MN, USA).

### Data handling and statistical analysis

From each patient there was one biopsy from the ileum and one or more colon biopsies. Therefore, for comparisons between non-inflamed and inflamed regions, we calculated the averages of the inflamed and of the non-inflamed colon biopsies for each patient, respectively. Differences between groups of patients or between ileum and colon biopsies were determined by Mann-Whitney U Test, and between different sites of the colon by Analysis of variance (ANOVA) with a Least Significant Difference (LSD) post-hoc test. The Spearman correlation coefficients were calculated for correlation assessments. Analyses were performed using SPSS (version 19.0; IBM Corporation, Armonk, NY, USA). Data are presented as mean ± standard error of the mean (SEM) and the significance levels were set to P<0.05 (*), 0.01 (**) or 0.001 (***).

## Results

### *PTPRZ1* expression is higher in colon compared to ileum of non-IBD subjects but is not altered in IBD patients compared to non-IBD subjects

**Fig 1:**
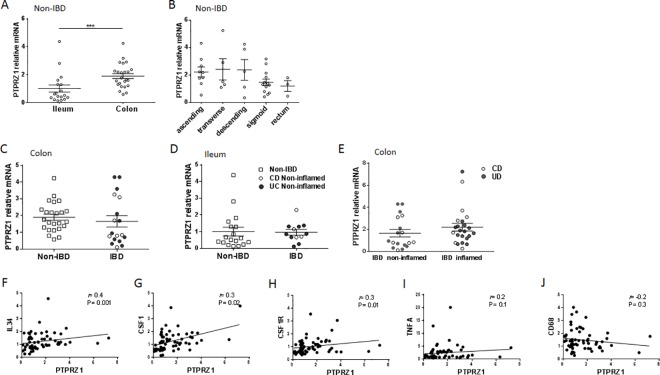
PTPRZ1 is differentially expressed in normal human ileum and colon but not regulated with inflammation. **(A)** *PTPRZ1* relative mRNA expression in ileum and colon, presented as the mean per colon sites for each non-IBD subjects. **(B)** *PTPRZ1* relative mRNA in different sites of the colon from non-IBD subjects. *PTPRZ1* relative mRNA expression in **(C)** colon and **(D)** ileum from non-IBD, IBD, UC and CD patients. Comparisons between ileum and colon were calculated using Mann–Whitney *U* tests, and between different sites of colon by ANOVA with post-hoc LSD tests. N = 18 for ileum, n = 24 for colon. Results are means ± SEM *P<0.05; **P<0.01; ***P<0.001. **(E)** *PTPRZ1* relative mRNA expression compared between non-inflamed and inflamed in IBD patients. *PTPRZ1* relative mRNA expression in colon presented as the mean per colon sites for each patient in IBD patients subdivided into CD and UC. Correlations of *PTPRZ1* with **(F)** *IL34*, **(G)** *CSF1*, **(H)** *CSF1R*, **(I)** *TNFA* and **(J)** *CD68* in IBD patients. Comparisons were evaluated using Mann–Whitney U tests. Correlations were assessed by Spearman’s correlation coefficients. N = 18 for non-inflamed IBD, n = 23 for inflamed IBD, n = 6 for non-inflamed CD, n = 6 for inflamed CD, n = 11 for non-inflamed UC, n = 16 for inflamed UC. Results are mean ± SEM *P<0.05; **P<0.01; ***P<0.001.

To assess whether the expression of *PTPRZ1* differs in normal human small intestine and colon, we analysed the mRNA expression in biopsies from ileum and colon of 27 controls without intestinal inflammation or any other pathological findings (S1 Table). The expression of *PTPRZ1* was significantly higher in colon compared to ileum (Fig 1A), whereas no differences between colon regions were observed (Fig 1B). No correlations between *PTPRZ1* and *IL34*, *CSF1*, *CSF1R*, *TNFA* or *CD68* were found in colon (S1A–S1E Fig) or ileum (S1F–S1J Fig) of non-IBD subjects. Given the observed expression of *PTPRZ1* in non-IBD ileum and colon we next investigated its expression in ileum and colon of IBD-patients. Compared to non-IBD subjects, there was no difference in *PTPRZ1* expression in non-inflamed regions of colon and ileum of IBD-patients (Fig 1C and 1D). Further, *PTPRZ1* expression was not altered with inflammation in IBD-patients (Fig 1E). However, in IBD-patients, *PTPRZ1* correlated to *IL34*, *CSF1* and *CSF1R* (Fig 1F–1H), but not to *TNFA* or *CD68* (Fig 1I and 1J).

### Diverse cellular expression of CSF-1R and PTPRZ1

**Fig 2:**
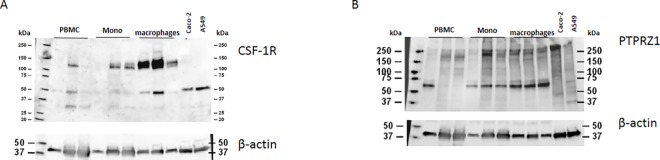
Expression of CSF-1R and PTPRZ1 in PBMCs, monocytes, macrophages and colonic epithelial cells. Immunoblotting of lysates from PBMCs, monocytes, CSF-1 monocyte derived and non-polarized macrophages, Caco-2 and A549 cells analysed for **(A)** CSF-1R and **(B)** PTPRZ1. β-actin was used as a loading control (10 and 15 μg protein per lane, respectively).

In light of previous work showing *CSF1R* gene expression in the gut [8], we assessed CSF-1R protein expression in PBMCs, monocytes, and macrophages of 3 healthy donors, in Caco-2 cells (a colonic epithelial cell line), and A549 cells by immunoblot analysis.We found heterogenous donor dependent CSF-1R expression in PBMCs and monocytes. The strongest CSF-1R expression is found in macrophages. In Caco-2 and A549 cells we detect a band of 52 kDa, the intracellular part of the receptor, (Fig 2A). We next investigated PTPRZ1 expression, and were not able to detect PTPRZ1 on RNA level in PBMCs, monocytes or macrophages. However we found very weak and donor dependent PTPRZ1 expression in PBMCs, monocytes, macrophages on protein level. Caco-2 cells and A549 cells were included as controls. The predicted band size is 255 kDa, and the smaller band size around 60 kDa is suspected to be intracellular receptor fragments (Fig 2B). To confirm that the weak staining shows expression of the protein of interest, different PTPRZ1 antibodies were used to detect PTPRZ1 in the positive controls Caco-2 and A549 cells (S3A and S3B Fig). Taken together, different molecular sizes of CSF-1R and PTPRZ1 were detected indicating that the receptors may have different modifications in different cells.

### Levels of cytokines and chemokines are altered by IL-34 and CSF-1 in PBMCs and monocytes but not in Caco-2 cells

**Fig 3:**
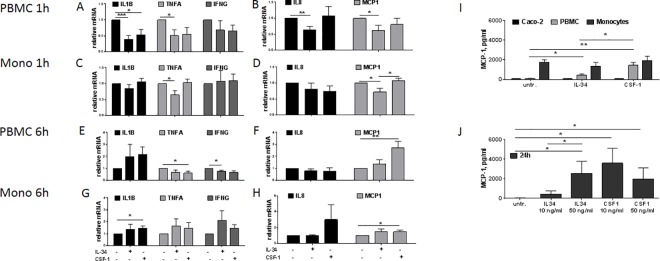
Regulation of pro—inflammatory cytokines and chemokines through IL-34 and CSF-1 in PBMCs and monocytes *IL1B*, *TNFA*, *IFNG*, *IL8* and *MCP1* relative mRNA expression in PBMCs. **(A, B)** and monocytes **(C, D)** stimulated with IL-34, CSF-1 for 1 h or left untreated were analysed by q-PCR and normalized to *GAPDH*. *IL1B*, *TNFA*, *IFNG*, *IL8* and *MCP1* relative mRNA expression from PBMCs **(E, F)** and monocytes **(G, H)** stimulated with IL-34, CSF-1 for 6 h or left untreated were analysed by q-PCR and normalized to *GAPDH*. Data represent mean + SEM, *P<0.05; **P<0.01; ***P<0.001, Student’s T-test, n = 5–6 donors. **(I)** Secreted MCP-1 in supernatants from Caco-2 cells, PBMCs and monocytes stimulated with IL-34 or CSF-1 for 24 h, as analysed by ELISA. Data represent mean + SEM, *P< 0.05; **P< 0.01; ***P< 0.001, Student’s T-test, n = 5–6 donors. **(J)** Secreted MCP-1 in supernatants from PBMCs stimulated with IL-34 or CSF-1 (10 or 50 ng/ml for 24h) were analysed by ELISA. Data represent mean + SEM, *P<0.05; **P<0.01; ***P<0.001, Student’s T-test, n = 6 donors.

We next analysed the capacity of IL-34 and CSF-1 to regulate the expression of cytokines and chemokines. Short-term (1h) stimulation of PBMCs with IL-34 or CSF-1 decreased *IL1B* expression (Fig 3A). In PBMCs, expression of *TNFA*, *IL8* and *MCP1* decreased after exposure to IL-34 (Fig 3A and 3B). In monocytes, *TNFA* and *MCP1* decreased after stimulation with IL-34 but not CSF-1. However, of note, expression of *MCP1* increased after stimulation with CSF-1 compared to IL-34, indicating differences in the stimulation pathway (Fig 3C and 3D). Longer (6h) stimulation of PBMCs resulted in decreased *TNFA* expression and increased *MCP1* expression with CSF-1 stimulation and decreased *IFNG* expression with IL-34 stimulation. Expression of *IL1B* showed a tendency to be up-regulated after stimulation with IL-34 or CSF-1, however this did not reach statistical significance (Fig 3E and 3F). 6h stimulation on monocytes resulted in increased *IL1B* and *MCP1* expression with CSF-1 stimulation, whereas expression was unaltered with IL-34 (Fig 3G and 3H). We also analysed the expression of *IL10* and *IL13*, and found decreased *IL13* expression with IL-34 stimulation in monocytes, whereas *IL10* and *IL13* expression was unchanged following IL-34 or CSF-1 stimulation in all other tested conditions (S2D–S2G Fig). We observed no changes in cytokines or chemokines in Caco-2 cells after stimulation with IL-34 or CSF-1 (S2A–S2C Fig).

We next analysed the MCP-1 protein secretion by Caco-2 cells, PBMCs, and monocytes after IL-34 or CSF-1 stimulation. MCP-1 secretion was induced by IL-34 or CSF-1 in PBMCs with more potent induction observed by CSF-1 compared to IL-34. No change in MCP-1 secretion was measured in Caco-2 cells or monocytes (Fig 3I). However, it should be noted that the baseline secretion of monocytes was comparable to the highest induced secretion from PBMCs. Subsequently, we analysed MCP-1 secretion in PBMCs stimulated with increasing concentrations of IL-34 or CSF-1. IL-34 induced MCP-1 in a dose-dependent manner whereas no difference was observed in response to CSF-1 (Fig 3J). Together these data show similarities and differences in the capacity of IL-34 and CSF-1 in the regulation of cytokines and chemokines in PBMCs and monocytes.

### CSF-1R regulate the expression of cytokines and chemokines in PBMCs and monocytes

**Fig 4:**
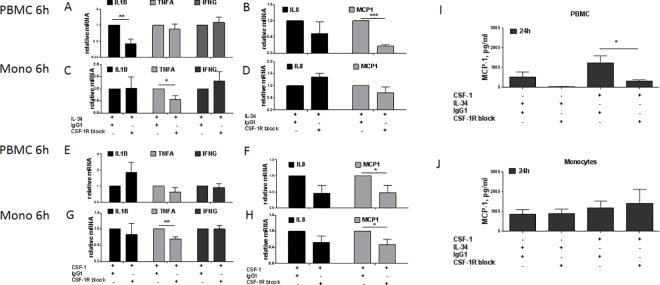
CSF-1R dependent regulation of pro-inflammatory cytokines and chemokines in PBMCs and monocytes *IL1B*, *TNFA*, *IFNG*, *IL8* and *MCP1* relative mRNA expression from PBMCs. **(A, B)** and monocytes **(C, D)** stimulated with IL-34, PBMCs **(E, F)** and monocytes **(G, H)** stimulated with CSF-1, after blocking CSF-1R for 6 h. IgG1 was used as a control. Gene expression was analysed by q-PCR and normalized to *GAPDH*. Data represent mean + SEM, *P<0.05; **P<0.01; ***P<0.001, Student’s T-test, n = 5–6 donors. (**I-J**) Secreted MCP-1 in supernatants from PBMCs **(I)** and monocytes **(J)** stimulated with IL-34 or CSF-1 after blocking CSF-1R for 24 h, IgG1 was used as a control, were analysed by ELISA. Data represent mean + SEM, *P<0.05; **P<0.01; ***P<0.001, Student’s T-test, n = 6 donors.

IL-34 and CSF-1 both signal through CSF-1R, but little is known about the consequences of blocking the receptor at the cellular level. We stimulated PBMCs and monocytes with IL-34 or CSF-1 and simultaneously blocked CSF-1R. In PBMCs, IL-34 stimulation together with CSF-1R blockade resulted in a decreased expression of *IL1B* and *MCP1*, (Fig 4A and 4B). In monocytes, the expression of *TNFA* after IL-34 stimulation and CSF-1R blocking decreased, no further regulation was measured (Fig 4C and 4D). CSF-1 stimulation together with CSF-1R blocking resulted in decreased *MCP1* expression in PBMCs (Fig 4F), interestingly, expression of *IL1B* showed the tendency of up-regulation which did not reach statistical significance (Fig 4E). Expression of *TNFA* and *MCP1* decreased in monocytes stimulated with CSF-1 while blocking the CSF-1R (Fig 4G and 4H). Consistently, MCP-1 secretion was reduced following stimulation with CSF-1 together with CSF-1R blocking in PBMCs (Fig 4I) but not in monocytes (Fig 4J) of matched donors. We observed no changes in *IL10* or *IL13* expression in PBMCs or monocytes after stimulation with IL-34 or CSF-1 while blocking the CSF-1R (S2H–S2K Fig). The data indicates MCP-1 regulation through CSF-1R in PBMCs, but not in monocytes, and *TNFA* regulation through CSF-1R in monocytes but not in PBMCs. The results are summarized in a model shown in S4 Fig.

### Cytokine expression in macrophages depends on polarization but not on CSF-1 and/or IL-34 induced differentiation

**Fig 5:**
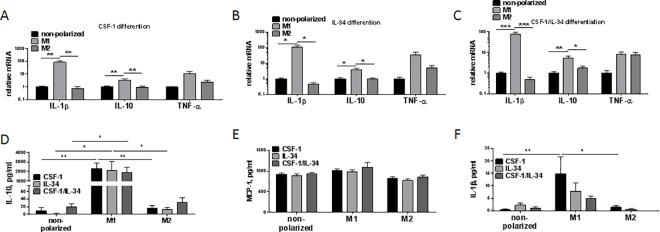
Diverse regulation of IL-10, IL-1β, TNF-α and MCP-1 in macrophages differentiated in the presence of IL-34 and CSF-1. **(A-C)** *IL1B*, *TNFA* and *IL10* mRNA expression in IL-34 and/or CSF-1 differentiated macrophages and polarized to an M1-like phenotype, an M2-like phenotype or non-polarized were analysed by q-PCR and normalized to *GAPDH*. Data represent mean + SEM, *P<0.05; **P<0.01; ***P<0.001, ANOVA, n = 5–6 donors. **(D-F)** Secreted IL-10, MCP-1 and IL-1β in the supernatant from IL-34 and/or CSF-1 differentiated macrophages and polarized to an M1-like phenotype, an M2-like phenotype or non-polarized were analysed by ELISA. Data represent mean + SEM, *P<0.05; **P< 0.01; ***P<0.001, ANOVA, n = 9 donors.

CSF-1 or IL-34 are known to induce macrophage differentiation. However, little is known about the effects of long-term CSF-1 or IL-34 treatment on cytokine profiles of macrophages. Therefore, we differentiated monocytes into macrophages by CSF-1 or IL-34 alone or in combination and polarized them into M1- or M2-like macrophages or left them non-polarized. *IL1B* and *IL10* expression increased after M1-like but were not regulated by M2-like polarization in all three differentiation conditions. The expression of *TNFA* slightly increased with M1-like polarization in the three differentiation conditions however, this not reach a statistical significance (Fig 5A–5C). On the protein level IL-10 was strongest induced in the M1-like polarized macrophages, independent of the differentiation whereas comparable levels were found in non- and M2-like polarized macrophages (Fig 5D). No differences in the release of MCP-1 was detected with the differentiation or polarization (Fig 5E). An increase in IL-1β protein secretion was measured in M1-like macrophages differentiated with CSF-1 whereas this was not observed following IL-34 differentiation or the combination of CSF-1 and IL-34 (Fig 5F).

## Discussion

Expression of the macrophage growth factors *IL34* and *CSF1* and their shared CSF-1 receptor (CSF-1R) was recently demonstrated in intestine and was also shown to be up-regulated with inflammation in IBD [8, 9, 20]. This, together with the identification of a new receptor for IL-34: receptor-type protein-tyrosine phosphatase ζ (PTPRZ1, RPTP-ζ) [11], prompted us to investigate the intestinal expression profile of *PTPRZ1*. We show higher *PTPRZ1* gene expression in colon compared to ileum in non-IBD subjects. We find no alteration in *PTPRZ1* expression with inflammation in IBD-patients. However, *PTPRZ1* positively correlates with *IL34*, *CSF1*, and *CSF1R* gene expression in IBD-patients. We also find differences in the expression patterns of CSF1R and PTPRZ1 in intestinal epithelial cells, PBMCs, monocytes, and macrophages. This is, to the best of our knowledge, the first study showing the expression of PTPRZ1 on the cellular level and in detail in non-IBD ileum and colon and various colonic segments. We also confirm the non-regulated *PTPRZ1* expression between inflamed and non-inflamed tissues of IBD patients [20].

We previously showed up-regulated intestinal *CSF1R* expression with inflammation in IBD patients [8]. Differences in expression and regulation of the two IL-34 receptors could be due to different signalling mechanisms of the receptors. PTPRZ1 has a constantly active phosphatase and ligand binding that results in an inhibited phosphatase activation, which in turn allows downstream tyrosine phosphorylation thus induced signal transduction [11]. Ligand binding to CSF-1R results in a receptor dimerization followed by a conformation change with autophosphorylation and activation of signal transduction pathways through STAT3 or JAK1 [21, 22]. PTPRZ1 and CSF-1R expression is reported for the brain [23, 24]. Whereas Ptprz1 is mainly expressed in the grey matter of the cortex, hippocampus and neurons, the expression of Csf-1r is described in the hippocampus, microglia and neurons [23, 24]. Interestingly, Fujikawa and colleagues showed that mice deficient for Ptprz1 were resistant to gastric ulcer induced by *Heliobacter pylori*. [25]. Little is known about PTPRZ1 function in other tissues or pathological conditions. To date, co-expression of these receptors is not reported but can be supposed based on their different expression patterns in the brain. IL-34 and PTPRZ1 are both expressed in regions of the forebrain and in mature neurons, however, only expression of PTPRZ1 is reported for the cerebellum [24, 26]. We found *CSF1R* and *PTPRZ1* expressed in intestinal tissue [8], monocytes, macrophages and in colon epithelial cells, however the expression in PBMCs was very week and donor dependent and will need to be confirmed in future studies. But these data let us surmise that co-expression of the receptors might be possible, which also needs to be investigated in further studies. We discovered enhanced *PTPRZ1* in the colon whereas *IL34* expression was higher in the ileum [8]. Of note, we were not able to detect PTPRZ1 expression in PBMCs, monocytes or macrophages on RNA level, whereas we could detect it in epithelial cells. This could be due to different reasons. Receptors can have a long turnover rate, in this case the existing protein is very stable, thus the amount of RNA needed is very low. Examples for a longer turnover are decribed in muscle cells, where the turnover can be detected on around day 8 to 13 [27]. The turnover in colon epithelial cells is around 3–5 days [28], which could explain the RNA expression in epithelial cells. Altenative RNA spicing products depending on the cell type, mainly described for the brain is another possible explanation [29], specially because PTPRZ1 expression is mainly described for the brain [23, 24]. Additionally PTPRZ1 studies, investigating expression on RNA and protein level in different cells are needed to identify possible mechanisms for this receptor.

Monocytes and monocyte-derived macrophages increase in numbers in the intestine in IBD and are described to be involved in the maintenance of intestinal inflammation [30–32]. Here we found differences in CSF-1R expression in monocytes, macrophages and PBMCs with, similar expression patterns. Expression of CSF-1R was strongest in macrophages and PTPRZ1 expression was detected mainly in monocytes and macrophages. The strongest expression of PTPRZ1 was measured in Caco-2 cells. Circulating monocytes and neutrophils, but not lymphocytes, were previously reported to express CSF-1R in whole blood [33]. Upon differentiation, we found increased CSF-1R expression. This is in agreement with the increase in receptor expression during myeloid cell differentiation [34]. CSF-1R and PTPRZ1 showed a stronger expression in monocytes and macrophages compared to PBMCs, whereas the highest expression of PTPRZ1 was detected in colonic epithelial cells. In this study, we used the Caco-2 cell line, to confirm this data, further studies investigating primary colon epithelial cells should be performed. Data on the expression of PTPRZ1 in monocytic THP-1 cells are conflicting where Booker and colleagues showed increased expression after ligand binding Seganily and colleagues reported no expression of PTPRZ1 in THP-1 cells [35, 36]. We used PBMCs and thus neutrophils were excluded by ficoll isolation. Besides monocytes and T- and B-lymphocytes, PBMCs include up to 15% NK cells [37, 38]. It is reported by Toh et al that monocytes but not lymphocytes express CSF-1R [33]. However, in their study CD3 was used as a marker to define lymphocytes and the majority of NK cells do not express CD3 [39]. Due to the reported increase in circulating and tissue NK cells in IBD [37], their receptor expression and potential regulation by IL-34 and CSF-1 in NK cells needs to be additionally investigated.

We further investigated differences in cytokines and chemokines in response to IL-34 and CSF-1. Short-term exposure (1h) to IL-34 and CSF-1 resulted in down-regulation of cytokines and chemokines in PBMCs and monocytes, where IL-34 regulated a higher number of genes compared to CSF-1, and in PBMCs more genes were regulated compared to monocytes, tested in our settings. The amino acid sequence of IL-34 and CSF-1 differs, unexpectedly the folded proteins show high structural similarities, thus may explain the stronger regulation by IL-34 after short incubation, while IL-34 is reported to bind more tightly but shorter compared to CSF-1 [7]. However, no difference in the induced signalling pathways is shown for the IL-34/CSF-1R complex compared to the CSF-1/CSF-1R complex, both ligands bind to the same receptor regions [7]. This may also explain, why after six hours incubation the main differences were observed in CSF-1, and not IL-34, stimulated cells. It has previously been reported that monocytes stimulated with physiological concentrations of 10 ng/ml CSF-1 show increased expression of IL-8, IL-6 and TNF-α [40], supporting our findings. Further, increased IL-1β, IFN-γ and MCP-1 expression was reported in human whole blood stimulated with IL-34 or CSF-1 [19]. This could be explained by the presence of NK cells and neutrophils in whole blood, known to express IFN-γ [37, 41], and by the presence of CSF-1R in neutrophils [33]. Expression of PTPRZ1 in circulating immune cells is not reported yet. We showed that mainly PBMCs and to a lesser degree monocytes were the main producer of chemokines and cytokines in response to IL-34 and CSF-1.

Blocking CSF-1R resulted in a down-regulation of MCP-1 with stronger effects in total PBMCs compared to monocytes, and *TNFA* specifically in monocytes but not in total PBMCs, independent of stimulation with IL-34 or CSF-1. The opposite regulation was observed for *IL1B*, IL-34 decreased whereas CSF-1 increased *IL1B* expression. These data provide insight that not all induced expression, e.g. IFN-γ, is reduced after blocking the CSF-1R, indicating the need to investigate blocking reagents also for PTPRZ1. These data indicate differences in cytokine and chemokine regulation by IL-34 and CSF-1 through CSF-1R. The results are combined in a model, including tendencies in the regulation by arrows in orange for slight down-regulation and light green arrows for a slight up-regulation. Clearly shown, PBMCs and monocytes react similarly and differently after the exposure to IL-34 or CSF-1, indicating different roles of the macrophage growth factors in the regulation of cytokines and chemokines (S3 Fig).

There is an increasing interest in IL-34 and CSF-1, where IL-34 overexpression was observed in inflammatory conditions including Sjögren’s syndrome, whereas overexpression of IL-34 and CSF-1 was reported for RA and IBD. [8, 9, 15, 16, 42] Interestingly, treatment with Infliximab reduces expression of IL-34 in RA patients. [17] In humans, IL-34 in serum can be detected in RA patients, [17, 43] however, we were unable to detect IL-34 in serum of non-IBD and IBD subjects (data not shown). Our data supports the hypothesis that the expression of PTPRZ1 and CSF-1R together with the macrophage growth factors IL-34 and CSF-1 are important during inflammation of the gastro-intestinal tract, and lead to the suggestion that IL-34 may act as a local marker and potential therapeutic target in IBD, however this has to be confirmed with functional data in future studies.

## Supporting Information

S1 Fig PTPRZ1 correlations in non-IBD patients Correlations of *PTPRZ1* gene expression with *IL34* **(A, F)**, *CSF1* **(B, G),** *CSF1R* **(C, H)**, *TNFA* **(D, I)** and *CD68* gene expression **(E, J)** in colon **(A–E)** and ileum **(F-J)** of non-IBD subjects. Correlations were assessed by Spearman’s correlation coefficients.
(TIF)

S2 Fig Regulation of pro- and anti-inflammatory cytokines and chemokines through IL-34 and CSF-1 in Caco-2 cells **(A-C)** *IL1B*, *TNFA*, *IFNG*, *IL10*, *IL13*, *IL8* and *MCP1* mRNA expression in Caco-2 cells stimulated with IL-34 and/or CSF-1 for 6 h or left untreated were analysed by q-PCR and normalized to *GAPDH*. Data represent mean + SEM, *P< 0.05; **P< 0.01; ***P< 0.001, Student’s T-test, n = 15. *IL10* and *IL13* relative mRNA expression in PBMCs **(D)** and monocytes **(E)** stimulated with IL-34, CSF-1 for 1 h or left untreated were analysed by q-PCR and normalized to *GAPDH*. *IL10* and *IL13* relative mRNA expression from PBMCs **(F)** and monocytes **(G)** stimulated with IL-34, CSF-1 for 6 h or left untreated were analysed by q-PCR and normalized to *GAPDH*. *IL10* and *IL13* relative mRNA expression from PBMCs **(H)** and monocytes **(I)** stimulated with IL-34, PBMCs **(J)** and monocytes **(K)** stimulated with CSF-1, after blocking CSF-1R for 6 h. IgG1 was used as a control and normalized to *GAPDH*. Data represent mean + SEM, *P<0.05; **P<0.01; ***P<0.001, Student’s T-test, n = 5–6 donors.
(TIF)

S3 Fig PTPRZ1 expression in Caco-2 and A549 cells Immunoblotting of lysates from Caco-2 and A549 cells analysed for (A) rabbit polyclonal anti-PTPRZ1 and (B) mouse monoclonal anti-PTPRZ1.
(TIF)

S4 Fig Scheme of the regulatory capacity of IL-34 and CSF-1 in PBMCs and monocytes Stimulation with IL-34 or CSF-1 have similar and different effects on the expression of pro-inflammatory cytokines and chemokines in PBMCs and monocytes after 6 h incubation: Stimulation with IL-34 decreased the expression *IFNG* in PBMCs, no further significant regulation was measured. However, tendencies of regulated expressions are marked with arrows in light green (up-regulation) or in orange (down-regulation). Stimulation with CSF-1 increased the expression of *IL1B* and *MCP1* in monocytes, in PBMCs *MCP1* was also up-regulated whereas the expression of *TNFA* was decreased. Blocking the CSF-1R and simultaneously stimulating with IL-34 resulted in down-regulation of *TNFA* in monocytes and likewise decreased expression of *IL1B* and *MCP1* in PBMCs. Stimulation with CSF-1 while blocking the CSF-1R resulted in a decreased expression of *TNFA* and *MCP1* in monocytes and *IL1B* and *MCP1* expression level in PBMCs.
(TIF)

S1 Table Characteristics of study participants (PDF)

S2 Table Sequence of forward and rivers primers used (PDF)
