# Supplementary figures and images for: Receptor-Type Protein-Tyrosine Phosphatase ζ and Colony Stimulating Factor-1 Receptor in the Intestine: Cellular Expression and Cytokine- and Chemokine Responses by Interleukin-34 and Colony Stimulating Factor-1
Source: PLoS One. 2016 Nov 29;11(11):e0167324. doi: 10.1371/journal.pone.0167324 (PMC5127567; doi:10.1371/journal.pone.0167324)

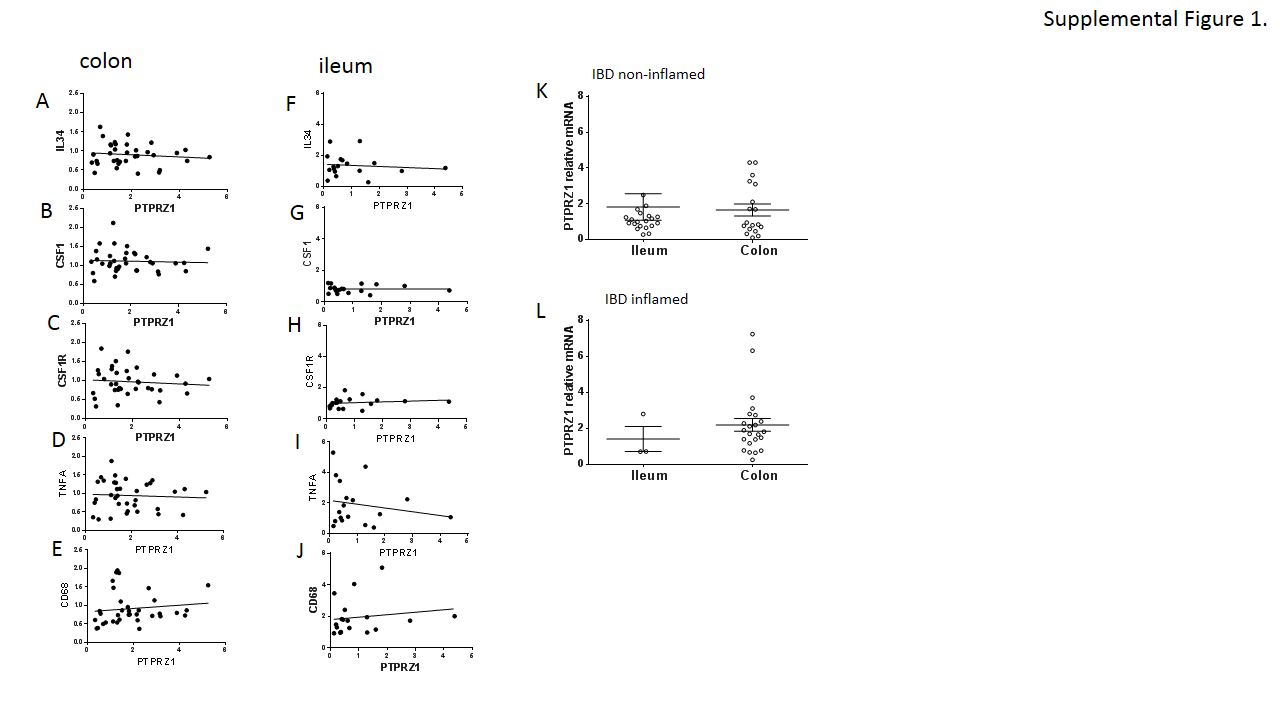

Supplement: S1 Fig — PTPRZ1 correlations in non-IBD patients Correlations of PTPRZ1 gene expression with IL34 (A, F), CSF1 (B, G), CSF1R (C, H), TNFA (D, I) and CD68 gene expression (E, J) in colon (A–E) and ileum (F-J) of non-IBD subjects. Correlations were assessed by Spearman’s correlation coefficients. (TIF) [file pone.0167324.s001.TIF]

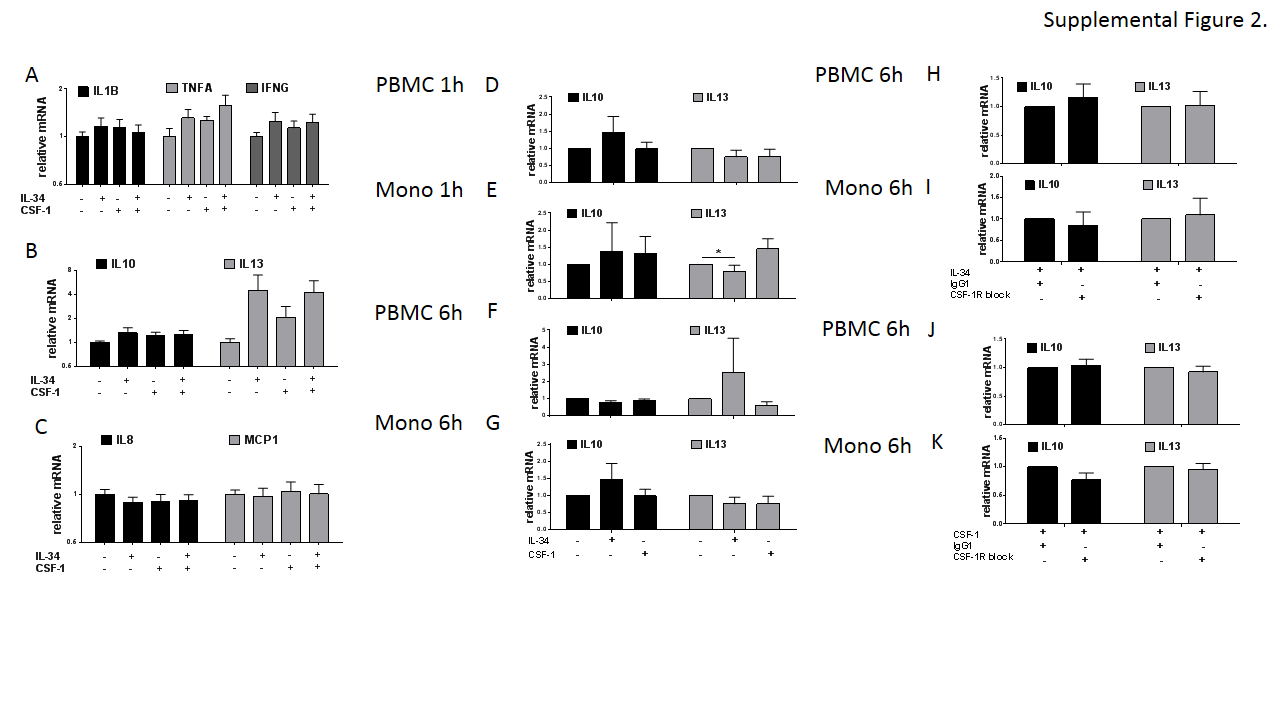

Supplement: S2 Fig — Regulation of pro- and anti-inflammatory cytokines and chemokines through IL-34 and CSF-1 in Caco-2 cells (A-C) IL1B, TNFA, IFNG, IL10, IL13, IL8 and MCP1 mRNA expression in Caco-2 cells stimulated with IL-34 and/or CSF-1 for 6 h or left untreated were analysed by q-PCR and normalized to GAPDH. Data represent mean + SEM, *P< 0.05; **P< 0.01; ***P< 0.001, Student’s T-test, n = 15. IL10 and IL13 relative mRNA expression in PBMCs (D) and monocytes (E) stimulated with IL-34, CSF-1 for 1 h or left untreated were analysed by q-PCR and normalized to GAPDH. IL10 and IL13 relative mRNA expression from PBMCs (F) and monocytes (G) stimulated with IL-34, CSF-1 for 6 h or left untreated were analysed by q-PCR and normalized to GAPDH. IL10 and IL13 relative mRNA expression from PBMCs (H) and monocytes (I) stimulated with IL-34, PBMCs (J) and monocytes (K) stimulated with CSF-1, after blocking CSF-1R for 6 h. IgG1 was used as a control and normalized to GAPDH. Data represent mean + SEM, *P<0.05; **P<0.01; ***P<0.001, Student’s T-test, n = 5–6 donors. (TIF) [file pone.0167324.s002.TIF]

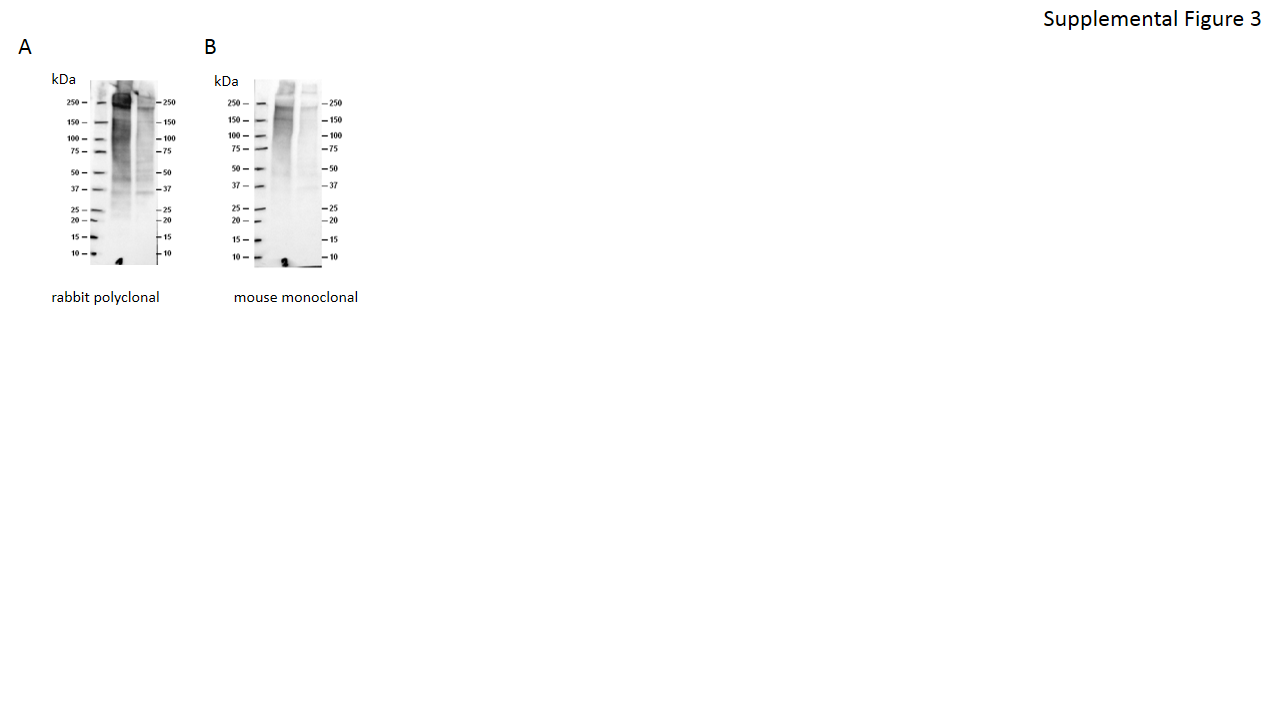

Supplement: S3 Fig — PTPRZ1 expression in Caco-2 and A549 cells Immunoblotting of lysates from Caco-2 and A549 cells analysed for (A) rabbit polyclonal anti-PTPRZ1 and (B) mouse monoclonal anti-PTPRZ1. (TIF) [file pone.0167324.s003.TIF]

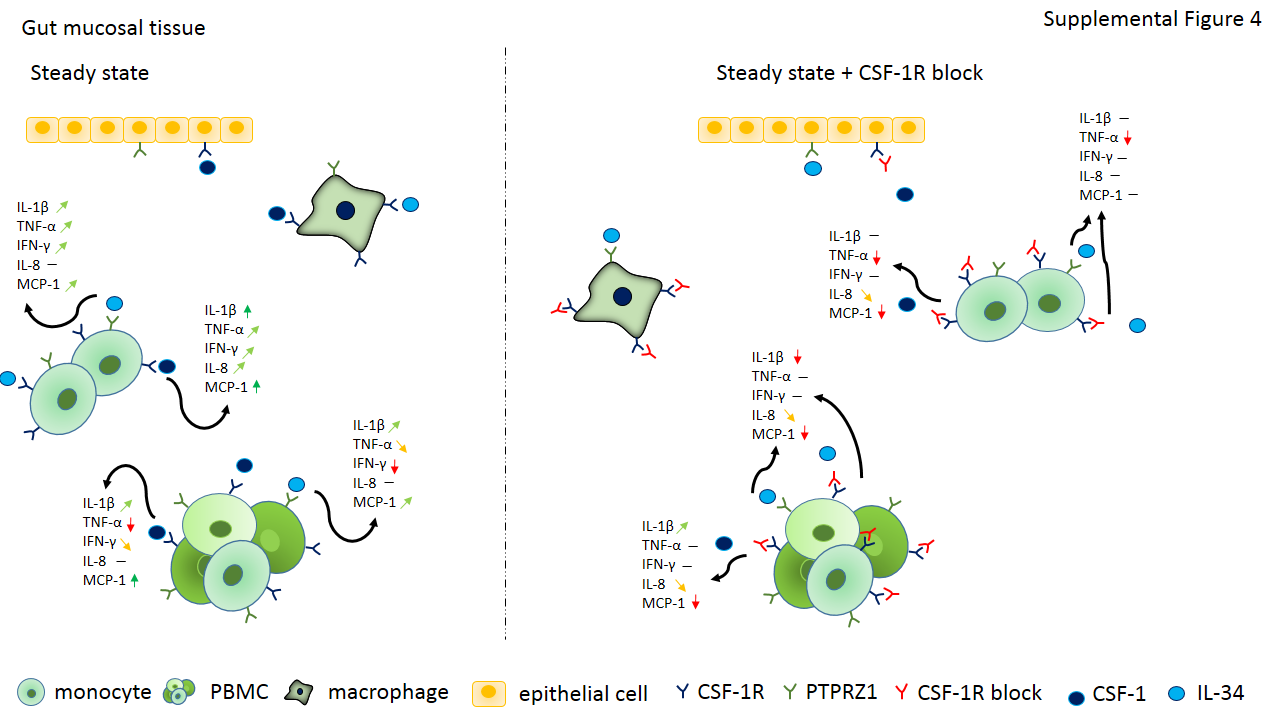

Supplement: S4 Fig — However, tendencies of regulated expressions are marked with arrows in light green (up-regulation) or in orange (down-regulation). Stimulation with CSF-1 increased the expression of IL1B and MCP1 in monocytes, in PBMCs MCP1 was also up-regulated whereas the expression of TNFA was decreased. Blocking the CSF-1R and simultaneously stimulating with IL-34 resulted in down-regulation of TNFA in monocytes and likewise decreased expression of IL1B and MCP1 in PBMCs. Stimulation with CSF-1 while blocking the CSF-1R resulted in a decreased expression of TNFA and MCP1 in monocytes and IL1B and MCP1 expression level in PBMCs. (TIF) [file pone.0167324.s004.TIF]
