# Supplementary material for: Receptor-Type Protein-Tyrosine Phosphatase ζ and Colony Stimulating Factor-1 Receptor in the Intestine: Cellular Expression and Cytokine- and Chemokine Responses by Interleukin-34 and Colony Stimulating Factor-1
Source: PLoS One. 2016 Nov 29;11(11):e0167324. doi: 10.1371/journal.pone.0167324 (PMC5127567; doi:10.1371/journal.pone.0167324)
Supplement: S1 Table — (PDF) [file pone.0167324.s005.pdf]

Supplemental. Table 1 Characteristics of study participants

	IBD (n=45)	Non-IBD (n=27)
**Disease** (CD/ UC/IBDU)	15/29/1	0
**Gender** (Male/ Female)	21/24	09/18
**Age** (years)[1]	38 (18-77)	47 (20-81)
**Immune-modulating drugs**		
Aminosalicylates	24	0
Thiopurines	13	0
Corticosteroids	14	0
Anti-TNF-α	4	1
Methotrexate	2	0
Tacrolimus	2	0
None	10	26

[1]Median (min-max)
