# Supplementary material for: Receptor-Type Protein-Tyrosine Phosphatase ζ and Colony Stimulating Factor-1 Receptor in the Intestine: Cellular Expression and Cytokine- and Chemokine Responses by Interleukin-34 and Colony Stimulating Factor-1
Source: PLoS One. 2016 Nov 29;11(11):e0167324. doi: 10.1371/journal.pone.0167324 (PMC5127567; doi:10.1371/journal.pone.0167324)
Supplement: S2 Table — (PDF) [file pone.0167324.s006.pdf]

	Forward	Reverse
**hIFNg**	**TCGGTAACTGACTTGAATGTCCA**	**TCGCTTCCCTGTTTTAGCTGC**
**hTNFa**	**GACAAGCCTGTAGCCCATGT**	**TCTCAGCTCCACGCCATT**
**hIL1b**	**TACCTGTCCTGCGTGTTGAA**	**TCTTTGGGTAATTTTTGGGATCT**
**hIL10**	**GACTTTAAGGGTTACCTGGGTTG**	**TCACATGCGCCTTGATGTCTG**
**hIL13**	**CCTCATGGCGCTTTTGTTGAC**	**TCTGGTTCTGGGTGATGTTGA**
**hMCP-1**	**AGTCTCTGCCGCCCTTCT**	**GTGACTGGGGCATTGATTG**
**hIL8**	**ACTGAGAGTGATTGAGAGTGGAC**	**AACCCTCTGCACCCAGTTTTC**
**hGAPDH**	**TCCCACTGGCGTCTTCACC**	**GGCAGAGATGATGACCCTTTT**
